# A physical and morphometric analysis of intraocular foreign bodies and their clinical correlation

**DOI:** 10.1007/s00417-025-07106-y

**Published:** 2026-01-09

**Authors:** Melih Parlak, Armin Wolf, Benjamin Mayer, Jens Ulrich Werner

**Affiliations:** 1 Department of Ophthalmology, Ulm University Medical School, Prittwitzstr. 43, Ulm, 89075 Germany; 2 Institute of Epidemiology and Medical Biometry, Ulm University, Ulm, Germany

**Keywords:** Intraocular foreign body, Ocular trauma, Penetrating injury, Vitrectomy

## Abstract

**Purpose:**

Penetrating injuries with intraocular foreign bodies (IOFB) remain a major cause of ocular morbidity worldwide. This study aimed to determine the physical and morphometric characteristics of IOFBs and investigate the correlation to the injury pattern.

**Methods:**

Intraocular foreign bodies retrieved between January 2018 and December 2023 were examined for dimension, weight, shape, and magneticity. In order to classify the shape of the IOFB, various indices were calculated. Clinical records, imaging data, and operative reports were retrospectively analyzed to determine the course of the incident, point of entry, and final intraocular position.

**Results:**

Fifty-four IOFBs were removed during the study period. Of these, 39 met the inclusion criteria. The majority of IOFBs were metal and magnetic (*n* = 32; 82%). Three IOFBs consisted of glass, two were made of wood and 2 eyelashes were extracted. The mean length, width and thickness were 4.95 mm, 1.70 mm and 0.86 mm, respectively. The average weight was 20.49 mg (0.2-112 mg; SD: 31 mg). The final intraocular location of foreign bodies was highly variable. With the exception of one case, all wire-shaped foreign bodies entered through the limbal cornea and penetrated into the suprachoroidal space. The variables of roundness, moment of inertia, length, circumference, and secondary moment of the major axis were identified as significant predictors of penetration depth.

**Conclusion:**

In order to break through the corneal or scleral rigidity, foreign bodies must have certain physical properties and sufficient kinetic energy upon impact. Specific foreign body shapes can also lead to specific injury patterns. These precise measurement data may be important for future studies in trauma prevention, materials research, or the development of ophthalmic instruments.

**Supplementary Information:**

The online version contains supplementary material available at 10.1007/s00417-025-07106-y.

## Key messages

*****What is known***:**

- Clinical characteristics and visual prognosis of penetrating ocular injuries involving an intraocular foreign body

*****What is new***:**

- Specific foreign body shapes can also lead to specific injury patterns
- The ocular penetration depth is influenced by the morphometric properties of intraocular foreign bodies

**Supplementary Information:**

The online version contains supplementary material available at 10.1007/s00417-025-07106-y.

## Introduction

Penetrating ocular injuries are a common and serious cause of morbidity worldwide, especially in the working population. In nearly one third of patients, the injury is associated with an intraocular foreign body (IOFB) [1, 2].

Due to the collagen-rich tissue architecture of the cornea and sclera, trajectory foreign bodies must have sufficient kinetic energy and shape to overcome tissue resistance at the point of impact. Similarly, unfavourable conditions of compressive force and shape must be present in puncture injuries [3].

While the epidemiology, pathogenesis of injury, and clinical characteristics have been extensively studied, there is limited data in the literature on the physical characteristics of IOFB.

This work examines in detail the physical and morphometric characteristics of intraocular foreign bodies in order to understand the unfavourable conditions of various injury patterns and to develop preventive measures if necessary.

## Materials and methods

This study was based on the intraocular foreign body database of the Ophthalmopathology Laboratory of the Department of Ophthalmology, Ulm University. Here, all foreign bodies are embedded in paraffin and archived after retrieval. The present study encompasses all intraocular foreign bodies that were surgically retrieved at the University Eye Hospital Ulm between January 2018 and December 2023. The study was approved by the institutional ethics committee and adhered to the tenets of the Declaration of Helsinki. Informed consent was waived due to the retrospective nature of the study. Epidemiologic data on the patient, the course of the accident, and the clinical findings were determined through a detailed review of the patient’s medical records. Available image and video data were also analyzed. Superficial foreign bodies that did not cause a thorough penetration of the cornea or sclera were excluded.

**Fig. 1:**
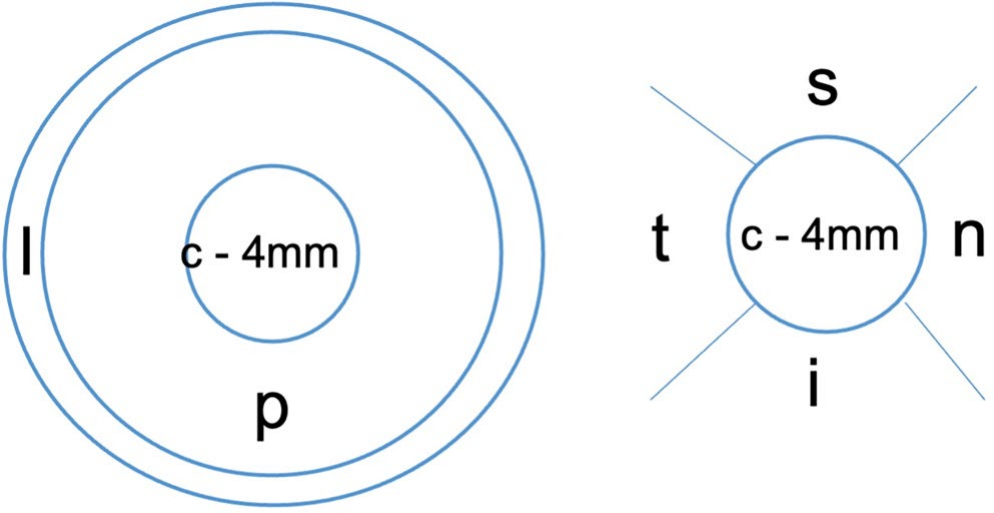
Classification of intraocular foreign body entry point: classification of cornea into central (4 mm, c), paracentral (4–9 mm, p) and limbal (9–11 mm, l). Additional sub-classification of quadrant in a right eye (s: superior; t: temporal; i: inferior; n: nasal)

The ocular entry point and the final intraocular position of the foreign body were determined based on patient records and available image data. For long foreign bodies, the point of deepest tissue penetration was recorded for the final intraocular position. The entry site was divided into 3 zones according to the Ocular Trauma Classification Group classification system (Zone I: Corneal up to and including the limbus; Zone 2: up to 5 mm posterior to the limbus; Zone III: injuries posterior to zone II). The corneal entry sites were again categorized into 3 subgroups: limbal, peripheral, and central. In addition, the entry site was radially divided into 4 quadrants (Fig. 1).

### Physical characteristics

After dissolving paraffin and sufficient drying time over 3 days, the foreign bodies were weighed on a closed balance with a resolution of 1 mg (Ohaus Adventurer AR1530, Ohaus Corp, New Jersey/USA). Three consecutive measurements were taken and the average value calculated. The weight of light foreign bodies less than 10 mg was also determined using a precision balance (Mettler S5, Geifensee).

To analyze the magnetic properties, all foreign bodies were tested for magnetic attraction in a glass container with a magnetic bar.

### Morphometric characteristics

All foreign bodies were digitally photographed with a digital microscope (Keyence VHX-7000, Keyence Corp, Osaka/Japan). After automatic segmentation, the following morphometric parameters were measured: area, circumference, maximum diameter, minimum diameter, roundness, circle equivalent diameter, moment of inertia (an objects resistance against a change in its angular velocity), secondary moment of inertia about the major and minor axis (an objects resistance being rotated about the major resp. minor axis) and width at maximum diameter.

An outside micrometer with a resolution of 0.01 mm was used to measure the thickness.

The foreign bodies were divided into 3 groups and analyzed separately according to the cause of the accident: High speed foreign bodies with trajectory, foreign bodies due to puncture injury, and cilia.

### Statistics

All data were analysed descriptively dependent on their scale level using Microsoft Excel and R for statistical computing (version 4.3.2). Frequencies were calculated for all categorical variables, whereas for all continuous variables the arithmetic mean, standard deviation (SD) and range were calculated. Graphical representation was based on bar charts and box-and-whisker plots. For foreign bodies with a trajectory, the penetration depth was classified into 3 levels: 1: suprachoroidal space, 2: anterior chamber and lens, and 3: vitreous body, retina. Following this ordinal scale level, possible predictors of penetration depth were exploratively analysed by means of a proportional odds model. The following predictors were examined with regard to penetration depth: weight, roundness, surface area, circumference, length, width, thickness, circle equivalent diameter, secondary moment of the minor and major axes, moment of inertia and width at maximum diameter. A *p*-value < 0.05 was considered statistically significant.

## Results

54 foreign bodies were extracted from our database during the study period. Of these, 39 met the inclusion criteria. The majority of patients were male (97%), and the mean age at injury was 42.3 years (2–74 years; SD: 15.4). All penetrating injuries were unilateral, with 14 cases (36%) affecting the right eye and 25 cases (64%) affecting the left eye. There were no ocular injuries with multiple foreign bodies.

The most common cause of injury was a hammer chisel trauma in 22 cases (56,4%). Other causes of injury were working mit rotating tools (saw, angle grinder, wire brush) (6 cases; 15.4%), falling (4 cases; 10.3%), drilling (1 case; 2.6%), impact by large objects (2 cases; 5.1%), other explosions (1 case; 4%) and passive injury of unknown origin (3 cases: 7.7%).

In 29 cases, the IOFB was retrieved during the initial surgery. In the remaining 10 patients, the foreign body was removed after reconstruction in the second surgery or at a later date in asymptomatic patients. The average time from injury to foreign body retrieval was 1.36 days (0–4 days). Excluded from this calculation were 2 cases where in one case the foreign body was intraocular for 4 years and in another case the remaining time was unclear.

- Ocular entry point and final position

The entry point showed a relatively even distribution. Ten foreign bodies entered the eye from the anterior sclera (zone 2), while 28 penetrated from the limbus and cornea (zone 1). Entry from the temporal quadrant was observed more frequently than in other quadrants. The distribution of the corneal entry point and the final intraocular position is shown in Table 1. While 10 IOFBs were retrieved from the anterior segment of the eye including the lens, 23 were removed from the vitreous or retina/choroid. All three foreign bodies, which were composed of glass, were successfully extracted from the anterior segment of the eye.

**Table 1:** Overview of the frequencies of the entry point and the final intraocular position (for one patient, the exact mechanism of injury and the point of entry were unknown)

Entry point	Frequency	Final intraocular position	Frequency
**Sclera**	10	Anterior Chamber	6
**Cornea**		Lens	4
Limbus	10	Suprachoroidal space	6
Central	6	Vitreous	6
Peripheral	12	Retina/Choroid	17
***Classified in quadrants***			
Superior	2		
Nasal	9		
Inferior	9		
Temporal	12		
Central	6

- Physical properties

The majority of IOFBs consisted of metal (n: 32; 82%) and were magnetic. Three IOFBs consisted of glass, two were made of wood and 2 eyelashes were extracted. The mean weight of IOFBs was 20.49 mg (0.2–112 mg; SD: 30.54). The foreign bodies varied widely in shape, size, and weight. With the exception of a single foreign body, the weight of all IOFB was found to be less than 100 mg.

- Morphometric properties

**Fig. 2:**
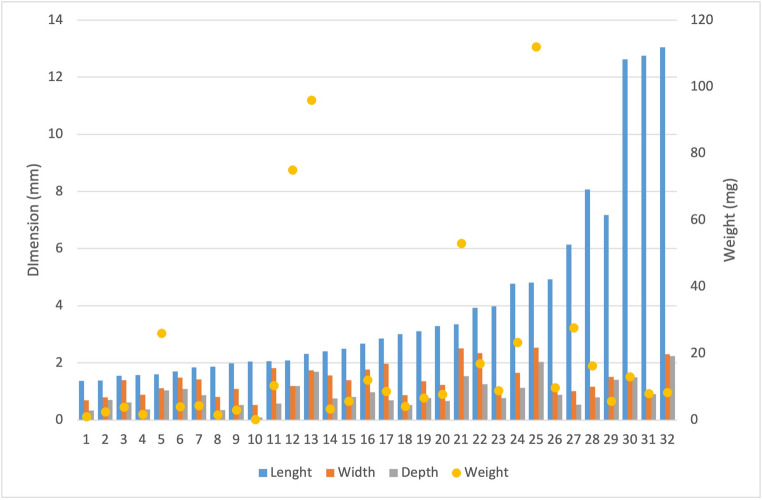
Distribution of dimension and weight

The distribution of dimensions and weights is shown in Fig. 2. Mean measurements of length, width and thickness of the IOFBs were 4.02±3.32 mm, 1.40±0.52 mm and 0.93±0.49 mm, respectively. The most common shape of the IOFBs was blade/cylinder shaped with a roundness between 0.2 and 0.7 (28; 72%). Four IOFBs had a spherical axial shape with a roundness greater than 0.7. The roundness of 4 metal wires and 2 lashes was less than 0.1. Further morphometric data are summarized in Table 2.

**Table 2:** Morphometric data of intraocular foreign bodies with and without trajectory

	IOFB with trajectory (*n* = 32)*	other IOFB (*n* = 7)*
Roundness	0.48 (0.05–0.82; 0.23)	0.36 (0.03–0.58; 0.24)
Area (mm2)	3.08 (0.7–7.47; 1.86)	3.51 (0.35–9.41; 3.51)
Circumference (mm)	10.6 (3.6–30.6; 6.9)	11.9 (5.24–19.02; 4.89)
Lenght (mm)	4.02 (1,37-13.05.05.05; 3.32)	4.32 (1.9–6.88; 1.74)
Width (mm)	1.4 (0.52–2.53; 0.52)	1.68 (0.87–2.96; 0.72)
Thickness	0.93 (0.09–2.24; 0.49)	1.26 (0.48–2.09; 0.50)
Circle equivalent diameter (mm)	1.89 (0.94–3.08; 0.60)	1.87 (0.67–3.46; 1.07)
Secondary moment of minor axis	25.6 (0.3–65.2; 17.1)	40.1 (15.3–94.6; 26.7)
Secondary moment of major axis	315 (28–1839; 508)	333 (68–831; 299)
Moment of inertia	0.64 (0.16–4.39; 1.10)	2.45 (0.18–8.09; 3.48)
Width at maximum diameter (mm)	1.45 (0.53–2.63; 0.53)	1.75 (1.04–3.11; 0.73)

*Mean (min-max; standard deviation)

- Penetration depth of IOFB with trajectory

**Fig. 3:**
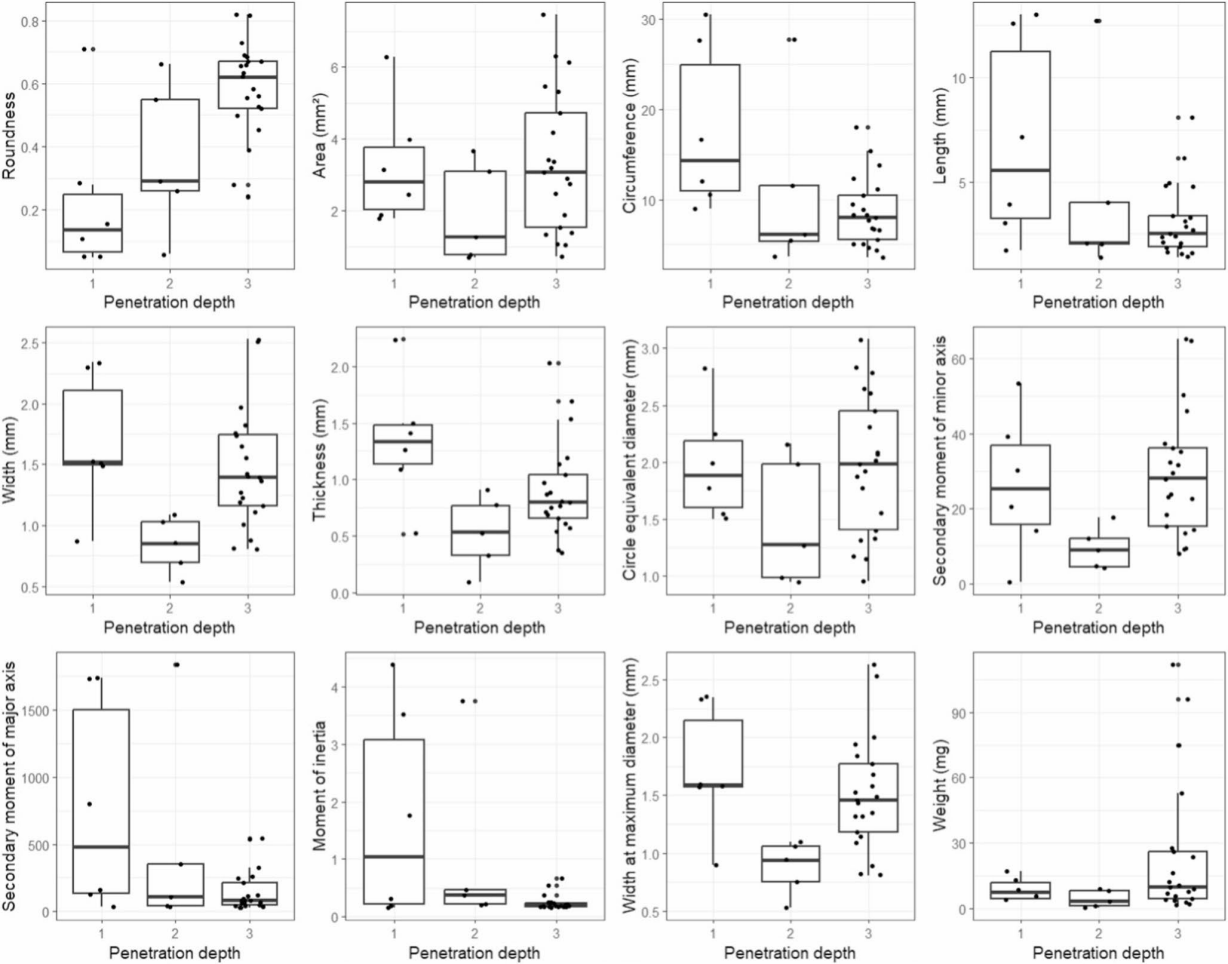
Graphical representation of morphometric data and penetration depth (with significant influence of roundness, length, moment of inertia, circumference and secondary moment of major axis in the ordinal logistic regression analysis)

The ordinal logistic regression analysis revealed that length, circumference, roundness, moment of inertia, and secondary moment of the major axis exerted a significant influence on the penetration depth. The distribution of the morphometric parameters and the results of the ordinal logistic regression analysis are summarized in Table 3; Fig. 3.

**Table 3:** Results of ordinal logistic regression analysis for penetration depth

Predictor	OR	95% CI	*p*-value
Roundness	***Not estimable***	***2.31–123554.5.31.5***	***0.001***
Area (mm²)	1.167	0.785–1.832	0.464
Circumference (mm)	***0.876***	***0.775–0.969***	***0.016***
Length (mm)	***0.783***	***0.615–0.963***	***0.026***
Width (mm)	1.216	0.306–5.520	0.785
Thickness (mm)	0.486	0.103–2.223	0.342
Circle equivalent diameter	1.503	0.457–5.355	0.508
Secondary moment of minor axis	1.034	0.987–1.095	0.196
Secondary moment of major axis	***0.998***	***0.996–1.000.996.000***	***0.024***
Moment of inertia	***0.378***	***0.135–0.744***	***0.015***
Width at maximum diameter (mm)	1.272	0.325–5.641	0.734
Weight (mg)	1.066	1.003–1.213	0.200

OR > 1 indicating increased risk for deeper penetration

## Discussion

Intraocular foreign bodies are serious ophthalmological emergencies that relatively often result in visual impairment. Extensive data on clinical characteristics and prognostic factors are already available, and further prognostic calculations, including artificial intelligence-based trauma scoring systems, are being evaluated [4]. In contrast, data on the physical properties of foreign bodies have only been studied in general terms. If we want to reduce the incidence of intraocular foreign bodies, we need to have a precise knowledge of the foreign bodies that can enter the eye.

Consistent with previous reports, the majority of patients were men of working age [5, 6]. The clinical characteristics and injury patterns correspond to the large service area of the University Eye Hospital, which treats mostly industrial and domestic injuries in Central Europe. Hammering and high-speed rotary tool accidents were the most common causes of accidents leading to penetrating eye injuries. There were no war injuries during the study period. Foreign bodies such as bullets that have penetrated the eye as a result of criminal violence were not examined in the study because the foreign bodies must be forwarded to the relevant authorities for forensic examination. The present study cohort must be interpreted accordingly. Penetrating injuries in children and projectile injuries caused by external force are more diverse and exhibit different characteristics [7].

The vast majority of foreign bodies were ferromagnetic and had a trajectory prior to impact. In order to overcome the relatively high tissue resistance of the cornea or sclera, IOFB must have certain properties in terms of mass, density, speed and shape [8]. In addition, the globe and the orbital fat tissue have a pressure-absorbing structure, which is particularly effective with a larger impact surface.

The descriptive data of our work show that the physical properties of foreign bodies with a trajectory have at least for industrial injuries a lower and upper threshold. While smaller foreign bodies may remain on the ocular surface, larger foreign bodies are more likely to cause a blunt trauma.

In a study conducted by Woodcock et al. [8], 69 foreign bodies were classified according to their mass and shape, and the correlation between these characteristics and their intraocular position was analyzed. While they found a significantly higher mass in foreign bodies that reached the posterior segment of the eye, the shape had no significant independent influence on the penetration depth. Contradictory data was reported in another study by Oeztas et al. [9]. In this cohort larger width, thickness and weight were associated with a higher incidence of retinal haemorrhage, vitreous haemorrhage and uveal prolapse, whereas longer foreign bodies were only more likely to be associated with hyphema. This suggests that the shape of the foreign body and a length index may play a crucial role in the event of impact.

In detail, we could show that the roundness, circumference, secondary moment of the major axis and the moment of inertia were predictive morphometric factors for the penetration depth. Especially the clinical characteristics of wire-shaped foreign bodies in our study are remarkable. Three out of four of these foreign bodies penetrated the globe through the limbal subsequently entering the suprachoroidal space, the 4th penetrated into the lens. Favourably, none of the wire-shaped foreign bodies reached the retina (supplemental content 1). In a case report by Kane et al. a similar patient was described where a metal wire penetrated from the limbus without damaging the lens and retina [10]. The authors hypothesize that ciliary body detachment smoothes the penetration pathway and prevents transretinal or transchoroidal penetration. It is obvious that the foreign body will pursue the least resistance during bulbus penetration. Instead of penetrating the cornea perpendicularly, penetration parallel to the collagen fibres seems conclusive for elongated foreign bodies. In addition, wire-shaped foreign bodies have a significantly higher moment of inertia and, compared to disc-shaped foreign bodies, rotate more slowly in their trajectory (conservation of angular momentum) [11]. In addition, it is possible that long foreign bodies are slowed down more during penetration due to their shape.

What are the typical characteristics of IOFBs with regard to penetration depth? Intraocular foreign bodies that enter the suprachoroidal space are typically highly irregular in shape, have a higher moment of inertia, are relatively light, and may be either short or long in length. Typical IOFBs of the anterior chamber or lens are narrow and thin, lightweight with a small surface area and have a small secondary moment of inertia as well as a small secondary moment of minor and major axis. IOFBs that enter the vitreous body or retina are quite round (small secondary moment of inertia, small secondary moment of minor axis). The surface area can be either small or large, resulting in a weight that can range from low to high. In light of these characteristics, the surgeon is able to prepare for the anticipated scenario during surgical treatment.

The course of the accident for wood and glass injuries differed from metal-containing foreign bodies with a trajectory. In this cohort, this type of foreign body injury was only caused by direct penetration, e.g. by falling, impact or breakage of a spectacle lens. In these cases, morphologically the sharpness and puncture force play a greater role than the weight and dimensions. These IOFBs were mainly extracted from the anterior segment.

The presence of intraocular cilia has been described in penetrating injuries and even after cataract surgery [12, 13]. In our cohort, 2 eyelashes were also found after penetrating eye injury. In rare instances, a cilium can be carried passively into the eye secondary to penetrating trauma. It is imperative that the primary penetrating object traverse the lid margin to facilitate the introduction of a cilium into the eye.

The present study is limited by the small number of cases included as a result of the exclusion criteria applied. Furthermore, the available data do not fully represent the actual cross-section, as very small foreign bodies measuring less than 1 mm can also be removed with an aspiration cannula or a vitrectome and are therefore not available for measurements. In our cohort, three foreign bodies exhibited an axial area of less than 1 mm², thereby corroborating the hypothesis that smaller foreign bodies with an appropriate density can penetrate the cornea or sclera. According to their low kinetic energy, 2 out of 3 of these foreign bodies were retrieved from the anterior chamber.

In conclusion, penetrating eye injuries with foreign bodies are complex conditions with frequently unpredictable outcomes and challenging surgical planning. The present study yielded evidence indicating the existence of specific injury patterns, which were found to be associated with particular physical and morphometric characteristics. These may be of importance in the initial surgical treatment or in the development of preventive measures.

## Supplementary Information

Below is the link to the electronic supplementary material.

Supplementary Material 1

## Data Availability

All data generated or analyzed during this study are included in this article. Further enquiries can be directed to the corresponding author.
